# Supplementary material for: Clinical impact of rapid molecular diagnostic tests in patients presenting with viral respiratory symptoms: A systematic literature review
Source: PLoS One. 2024 Jun 13;19(6):e0303560. doi: 10.1371/journal.pone.0303560 (PMC11175541; doi:10.1371/journal.pone.0303560)
Supplement: S1 Table — (PDF) [file pone.0303560.s001.pdf]

Embase search strategy

Embase <1974 to 2023 April 18>; Search executed: April 19, 2023

Line	Search terms	Hits
**1**	exp point-of-care testing/	20,870
**2**	exp molecular diagnostic techniques/	28,437
**3**	exp COVID-19 nucleic acid testing/	1,219
**4**	(((rapid or "point of care" or POC or "near patient" or bedside or "real time") adj4 (test* or detect* or assay* or diagnos*)) or radt or ridt or naat).mp.	214,266
**5**	or/1-4	240,818
**6**	exp influenza A virus/ or exp influenza B virus/ or exp influenza, human/	117,362
**7**	(influenza or flu).mp.	201,719
**8**	exp respiratory syncytial viruses/	9,148
**9**	(respiratory syncytial virus* or RSV).mp.	31,996
**10**	exp SARS-CoV-2/ or exp COVID-19/	370,955
**11**	(nCoV* or 2019nCoV or 19nCoV or COVID19* or COVID or SARS-COV-2 or SARSCOV-2 or SARS-COV2 or SARSCOV2 or SARS coronavirus 2 or Severe Acute Respiratory Syndrome Coronavirus 2 or Severe Acute Respiratory Syndrome Corona Virus 2 or ((new or novel or "19" or "2019" or Wuhan or Hubei or China or Chinese) adj3 (coronavirus* or corona virus* or betacoronavirus* or CoV or HCoV))).mp. [mp=title, abstract, heading word, drug trade name, original title, device manufacturer, drug manufacturer, device trade name, keyword heading word, floating subheading word, candidate term word]	450,262
**12**	exp respiratory tract infections/	484,958
**13**	(respiratory adj3 (infect* or virus* or viral)).mp.	186,834
**14**	or/6-13	1,022,035
**15**	5 and 14	24,542
**16**	Clinical Trial/	1,073,377
**17**	Randomized Controlled Trial/	780,006
**18**	controlled clinical trial/	468,985
**19**	multicenter study/	374,023
**20**	Phase 3 clinical trial/	69,056
**21**	Phase 4 clinical trial/	5,401
**22**	exp RANDOMIZATION/	99,164
**23**	Single Blind Procedure/	51,450
**24**	Double Blind Procedure/	209,386
**25**	Crossover Procedure/	74,879
**26**	PLACEBO/	401,601
**27**	randomi?ed controlled trial$.tw.	323,088
**28**	rct.tw.	53,131
**29**	(random$ adj2 allocat$).tw.	54,355
**30**	single blind$.tw.	31,526
**31**	double blind$.tw.	244,249
**32**	((treble or triple) adj blind$).tw.	1,857
**33**	placebo$.tw.	366,398
**34**	Prospective Study/	868,952
**35**	single arm.tw.	27,574
**36**	(Phase II or Phase 2).tw.	161,964

Line	Search terms	Hits
**37**	Phase 2 clinical trial/	106,318
**38**	or/16-37	3,017,930
**39**	Clinical study/	162,739
**40**	Case control study/	204,738
**41**	Family study/	25,765
**42**	Longitudinal study/	191,913
**43**	Retrospective study/	1,451,616
**44**	Prospective study/	868,952
**45**	Cohort analysis/	1,021,597
**46**	(Cohort adj (study or studies)).mp.	472,672
**47**	(Case control adj (study or studies)).tw.	168,004
**48**	(follow up adj (study or studies)).tw.	73,485
**49**	(observational adj (study or studies)).tw.	251,398
**50**	(epidemiologic$ adj (study or studies)).tw.	122,129
**51**	(cross sectional adj (study or studies)).tw.	334,477
**52**	or/39-51	3,887,755
**53**	socioeconomics/	159,281
**54**	exp Quality of Life/	641,166
**55**	quality of life.ti,kf.	180,104
**56**	((instrument or instruments) adj3 quality of life).ab.	5,423
**57**	Quality-Adjusted Life Year/	34,967
**58**	quality adjusted life.ti,ab,kf.	26,734
**59**	(qaly* or qald* or qale* or qtime* or life year or life years).ti,ab,kf.	44,591
**60**	disability adjusted life.ti,ab,kf.	6,349
**61**	daly*.ti,ab,kf.	6,126
**62**	(sf36 or sf 36 or short form 36 or shortform 36 or short form36 or shortform36 or sf thirtysix or sfthirtysix or sfthirty six or sf thirty six or shortform thirtysix or shortform thirty six or short form thirtysix or short form thirty six).ti,ab,kf.	49,946
**63**	(sf6 or sf 6 or short form 6 or shortform 6 or sf six or sfsix or shortform six or short form six or shortform6 or short form6).ti,ab,kf.	2,925
**64**	(sf8 or sf 8 or sf eight or sfeight or shortform 8 or shortform 8 or shortform8 or short form8 or shortform eight or short form eight).ti,ab,kf.	1,028
**65**	(sf12 or sf 12 or short form 12 or shortform 12 or short form12 or shortform12 or sf twelve or sftwelve or shortform twelve or short form twelve).ti,ab,kf.	12,254
**66**	(sf16 or sf 16 or short form 16 or shortform 16 or short form16 or shortform16 or sf sixteen or sfsixteen or shortform sixteen or short form sixteen).ti,ab,kf.	72
**67**	(sf20 or sf 20 or short form 20 or shortform 20 or short form20 or shortform20 or sf twenty or sftwenty or shortform twenty or short form twenty).ti,ab,kf.	522
**68**	(hql or hqol or h qol or hrqol or hr qol).ti,ab,kf.	38,970
**69**	(hye or hyes).ti,ab,kf.	169
**70**	(health* adj2 year* adj2 equivalent*).ti,ab,kf.	56
**71**	(pqol or qls).ti,ab,kf.	744
**72**	(quality of wellbeing or quality of well being or index of wellbeing or index of well being or qwb).ti,ab,kf.	887
**73**	exp "named inventories, questionnaires and rating scales"/	1,934,073
**74**	nottingham health profile*.ti,ab,kf.	1,665
**75**	sickness impact profile.ti,ab,kf.	1,285

Line	Search terms	Hits
**76**	health status indicator/	3,443
**77**	(health adj3 (utilit* or status)).ti,ab,kf.	120,253
**78**	(utilit* adj3 (valu* or measur* or health or life or estimat* or elicit* or disease or score* or weight)).ti,ab,kf.	25,408
**79**	(preference* adj3 (valu* or measur* or health or life or estimat* or elicit* or disease or score* or instrument or instruments)).ti,ab,kf.	18,988
**80**	disutilit*.ti,ab,kf.	1,232
**81**	rosser.ti,ab,kf.	143
**82**	willingness to pay.ti,ab,kf.	12,970
**83**	standard gamble*.ti,ab,kf.	1,213
**84**	(time trade off or time tradeoff).ti,ab,kf.	2,388
**85**	tto.ti,ab,kf.	2,211
**86**	(hui or hui1 or hui2 or hui3).ti,ab,kf.	3,067
**87**	(eq or euroqol or euro qol or eq5d or eq 5d or euroqual or euro qual).ti,ab,kf.	37,661
**88**	duke health profile.ti,ab,kf.	118
**89**	functional status questionnaire.ti,ab,kf.	173
**90**	dartmouth coop functional health assessment*.ti,ab,kf.	14
**91**	or/53-90	2,649,775
**92**	Socioeconomics/	159,281
**93**	Cost benefit analysis/	93,656
**94**	Cost effectiveness analysis/	179,275
**95**	Cost of illness/	21,139
**96**	Cost control/	75,816
**97**	Economic aspect/	123,627
**98**	Financial management/	120,686
**99**	Health care cost/	221,860
**100**	Health care financing/	13,841
**101**	Health economics/	35,505
**102**	Hospital cost/	25,161
**103**	(fiscal or financial or finance or funding).tw.	285,618
**104**	Cost minimization analysis/	3,971
**105**	(cost adj estimate$).mp.	4,181
**106**	(cost adj variable$).mp.	321
**107**	(unit adj cost$).mp.	5,520
**108**	or/92-107	1,106,458
**109**	38 or 52 or 91 or 108	8,029,287
**110**	Case Study/	97,729
**111**	case report.tw.	529,094
**112**	abstract report/ or letter/	1,311,474
**113**	Conference proceeding.pt.	0
**114**	Conference abstract.pt.	4,735,223
**115**	Editorial.pt.	771,659
**116**	Letter.pt.	1,298,717
**117**	Note.pt.	935,201
**118**	or/110-117	8,288,671
**119**	109 not 118	5,833,538
**120**	15 and 119	6,008
**121**	limit 120 to yr=2010 - current	5,425
**122**	limit 121 to english	5,301
