# Supplementary material for: Clinical impact of rapid molecular diagnostic tests in patients presenting with viral respiratory symptoms: A systematic literature review
Source: PLoS One. 2024 Jun 13;19(6):e0303560. doi: 10.1371/journal.pone.0303560 (PMC11175541; doi:10.1371/journal.pone.0303560)
Supplement: S2 Table — (PDF) [file pone.0303560.s002.pdf]

MEDLINE search strategy

Ovid MEDLINE(R) and Epub Ahead of Print, In-Process & Other Non-Indexed Citations and Daily

1946 to 2023 April 18; Executed on 2023 April 19

Line	Search terms	Hits
1	exp point-of-care testing/	4,085
2	exp molecular diagnostic techniques/	20,977
3	exp COVID-19 nucleic acid testing/	1,618
4	(((rapid or "point of care" or POC or "near patient" or bedside or "real time") adj4 (test* or detect* or assay* or diagnos*)) or radt or ridt or naat).mp.	152,515
5	or/1-4	171,387
6	exp influenza A virus/ or exp influenza B virus/ or exp influenza, human/	81,882
7	(influenza or flu).mp.	137,415
8	exp respiratory syncytial viruses/	10,482
9	(respiratory syncytial virus* or RSV).mp.	22,836
10	exp SARS-CoV-2/ or exp COVID-19/	225,815
11	(nCoV* or 2019nCoV or 19nCoV or COVID19* or COVID or SARS-COV-2 or SARSCOV-2 or SARS-COV2 or SARSCOV2 or SARS coronavirus 2 or Severe Acute Respiratory Syndrome Coronavirus 2 or Severe Acute Respiratory Syndrome Corona Virus 2 or ((new or novel or "19" or "2019" or Wuhan or Hubei or China or Chinese) adj3 (coronavirus* or corona virus* or betacoronavirus* or CoV or HCoV))).mp. [mp=title, book title, abstract, original title, name of substance word, subject heading word, floating sub-heading word, keyword heading word, organism supplementary concept word, protocol supplementary concept word, rare disease supplementary concept word, unique identifier, synonyms, population supplementary concept word, anatomy supplementary concept word]	353,394
12	exp respiratory tract infections/	602,303
13	(respiratory adj3 (infect* or virus* or viral)).mp.	107,768
14	or/6-13	847,813
15	5 and 14	17,857
16	Randomized Controlled Trials as Topic/	161,669
17	randomized controlled trial/	591,286
18	Random Allocation/	106,920
19	Double Blind Method/	174,934
20	Single Blind Method/	32,645
21	clinical trial/	537,788
22	clinical trial, phase i.pt.	24,825
23	clinical trial, phase ii.pt.	39,572
24	clinical trial, phase iii.pt.	21,626
25	clinical trial, phase iv.pt.	2,405
26	controlled clinical trial.pt.	95,277
27	randomized controlled trial.pt.	591,286
28	multicenter study.pt.	332,947
29	clinical trial.pt.	537,788
30	exp Clinical Trials as topic/	381,787

Line	Search terms	Hits
**31**	(clinical adj trial$).tw.	470,965
**32**	((singl$ or doubl$ or treb$ or tripl$) adj (blind$3 or mask$3)).tw.	196,198
**33**	PLACEBOS/	35,926
**34**	placebo$.tw.	245,274
**35**	randomly allocated.tw.	35,920
**36**	(allocated adj2 random$).tw.	39,654
**37**	single arm.tw.	12,570
**38**	or/16-37	1,903,950
**39**	Epidemiologic studies/	9,304
**40**	exp case control studies/	1,408,387
**41**	exp cohort studies/	2,471,518
**42**	Case control.tw.	152,068
**43**	(cohort adj (study or studies)).tw.	308,521
**44**	Cohort analy$.tw.	11,529
**45**	(Follow up adj (study or studies)).tw.	55,839
**46**	(observational adj (study or studies)).tw.	157,417
**47**	Longitudinal.tw.	317,187
**48**	Retrospective.tw.	729,358
**49**	Cross sectional.tw.	501,643
**50**	Cross-sectional studies/	463,788
**51**	or/39-50	3,745,717
**52**	"Value of Life"/	5,806
**53**	Quality of Life/	264,029
**54**	quality of life.ti,kf.	113,652
**55**	((instrument or instruments) adj3 quality of life).ab.	3,886
**56**	Quality-Adjusted Life Years/	15,555
**57**	quality adjusted life.ti,ab,kf.	17,091
**58**	(qaly* or qald* or qale* or qtime* or life year or life years).ti,ab,kf.	27,562
**59**	disability adjusted life.ti,ab,kf.	5,157
**60**	daly*.ti,ab,kf.	4,661
**61**	(sf36 or sf 36 or short form 36 or shortform 36 or short form36 or shortform36 or sf thirtysix or sfthirtysix or sfthirty six or sf thirty six or shortform thirtysix or shortform thirty six or short form thirtysix or short form thirty six).ti,ab,kf.	30,330
**62**	(sf6 or sf 6 or short form 6 or shortform 6 or sf six or sfsix or shortform six or short form six or shortform6 or short form6).ti,ab,kf.	2,601
**63**	(sf8 or sf 8 or sf eight or sfeight or shortform 8 or shortform 8 or shortform8 or short form8 or shortform eight or short form eight).ti,ab,kf.	615
**64**	(sf12 or sf 12 or short form 12 or shortform 12 or short form12 or shortform12 or sf twelve or sftwelve or shortform twelve or short form twelve).ti,ab,kf.	7,534
**65**	(sf16 or sf 16 or short form 16 or shortform 16 or short form16 or shortform16 or sf sixteen or sfsixteen or shortform sixteen or short form sixteen).ti,ab,kf.	40
**66**	(sf20 or sf 20 or short form 20 or shortform 20 or short form20 or shortform20 or sf twenty or sftwenty or shortform twenty or short form twenty).ti,ab,kf.	452
**67**	(hql or hqol or h qol or hrqol or hr qol).ti,ab,kf.	23,616
**68**	(hye or hyes).ti,ab,kf.	76
**69**	(health* adj2 year* adj2 equivalent*).ti,ab,kf.	48
**70**	(pqol or qls).ti,ab,kf.	456

Line	Search terms	Hits
**71**	(quality of wellbeing or quality of well being or index of wellbeing or index of well being or qwb).ti,ab,kf.	703
**72**	nottingham health profile*.ti,ab,kf.	1,231
**73**	sickness impact profile.ti,ab,kf.	1,093
**74**	exp health status indicators/	341,521
**75**	(health adj3 (utilit* or status)).ti,ab,kf.	90,657
**76**	(utilit* adj3 (valu* or measur* or health or life or estimat* or elicit* or disease or score* or weight)).ti,ab,kf.	15,640
**77**	(preference* adj3 (valu* or measur* or health or life or estimat* or elicit* or disease or score* or instrument or instruments)).ti,ab,kf.	14,160
**78**	disutilit*.ti,ab,kf.	608
**79**	rosser.ti,ab,kf.	109
**80**	willingness to pay.ti,ab,kf.	8,408
**81**	standard gamble*.ti,ab,kf.	912
**82**	(time trade off or time tradeoff).ti,ab,kf.	1,649
**83**	tto.ti,ab,kf.	1,386
**84**	(hui or hui1 or hui2 or hui3).ti,ab,kf.	1,940
**85**	(eq or euroqol or euro qol or eq5d or eq 5d or euroqual or euro qual).ti,ab,kf.	22,165
**86**	duke health profile.ti,ab,kf.	92
**87**	functional status questionnaire.ti,ab,kf.	131
**88**	dartmouth coop functional health assessment*.ti,ab,kf.	14
**89**	or/52-88	742,585
**90**	Economics/	27,499
**91**	"costs and cost analysis"/	51,270
**92**	Cost allocation/	2,018
**93**	Cost-benefit analysis/	92,179
**94**	Cost control/	21,661
**95**	Cost savings/	12,697
**96**	Cost of illness/	31,416
**97**	Cost sharing/	2,733
**98**	"deductibles and coinsurance"/	1,850
**99**	Medical savings accounts/	547
**100**	Health care costs/	44,018
**101**	Direct service costs/	1,217
**102**	Drug costs/	17,355
**103**	Employer health costs/	1,097
**104**	Hospital costs/	11,933
**105**	Health expenditures/	23,862
**106**	Capital expenditures/	2,001
**107**	Value of life/	5,806
**108**	exp economics, hospital/	25,697
**109**	exp economics, medical/	14,386
**110**	Economics, nursing/	4,013
**111**	Economics, pharmaceutical/	3,098
**112**	exp "fees and charges"/	31,338
**113**	exp budgets/	14,099
**114**	(low adj cost).mp.	85,041
**115**	(high adj cost).mp.	19,418
**116**	(health?care adj cost$).mp.	16,156
**117**	(fiscal or funding or financial or finance).tw.	194,335
**118**	(cost adj estimate$).mp.	2,708
**119**	(cost adj variable).mp.	50

Line	Search terms	Hits
**120**	(unit adj cost$).mp.	3,092
**121**	(economic$ or pharmacoeconomic$ or price$ or pricing).tw.	400,540
**122**	or/90-121	916,642
**123**	38 or 51 or 89 or 122	6,184,568
**124**	case report.tw.	390,643
**125**	letter/	1,213,841
**126**	historical article/	369,202
**127**	or/124-126	1,954,909
**128**	123 not 127	6,025,437
**129**	15 and 128	5,236
**130**	limit 129 to yr=2010 - current	4,514
**131**	limit 130 to english	4,395
