# Supplementary material for: Clinical impact of rapid molecular diagnostic tests in patients presenting with viral respiratory symptoms: A systematic literature review
Source: PLoS One. 2024 Jun 13;19(6):e0303560. doi: 10.1371/journal.pone.0303560 (PMC11175541; doi:10.1371/journal.pone.0303560)
Supplement: S3 Table — (PDF) [file pone.0303560.s003.pdf]

EconLit search strategy

Ovid EconLit 1886 to 2023 April 13; Executed on 19 April 2023

Line	Search terms	Hits
**1**	(((rapid or "point of care" or POC or "near patient" or bedside or "real time") adj4 (test* or detect* or assay* or diagnos*)) or radt or ridt or naat).mp.	165
**2**	(influenza or flu).mp.	673
**3**	(respiratory syncytial virus* or RSV).mp.	16
**4**	(nCoV* or 2019nCoV or 19nCoV or COVID19* or COVID or SARS-COV-2 or SARSCOV-2 or SARS-COV2 or SARSCOV2 or SARS coronavirus 2 or Severe Acute Respiratory Syndrome Coronavirus 2 or Severe Acute Respiratory Syndrome Corona Virus 2 or ((new or novel or "19" or "2019" or Wuhan or Hubei or China or Chinese) adj3 (coronavirus* or corona virus* or betacoronavirus* or CoV or HCoV))).mp. [mp=heading words, abstract, title, country as subject]	10,656
**5**	(respiratory adj3 (infect* or virus* or viral)).mp.	103
**6**	or/2-5	11,332
**7**	1 and 6	11
**8**	limit 7 to yr=2010 - current	11
**9**	limit 8 to english	11
