# Supplementary material for: Clinical impact of rapid molecular diagnostic tests in patients presenting with viral respiratory symptoms: A systematic literature review
Source: PLoS One. 2024 Jun 13;19(6):e0303560. doi: 10.1371/journal.pone.0303560 (PMC11175541; doi:10.1371/journal.pone.0303560)
Supplement: S4 Table — (PDF) [file pone.0303560.s004.pdf]

CENTRAL search strategy

EBM Reviews - Cochrane Central Register of Controlled Trials - March 2023; Executed on 19 April 2023

Line	Search terms	Hits
**1**	exp point-of-care testing/	189
**2**	exp molecular diagnostic techniques/	144
**3**	exp nucleic acid amplification techniques/	3,360
**4**	exp COVID-19 nucleic acid testing/	30
**5**	(((rapid or "point of care" or POC or "near patient" or bedside or "real time") adj4 (test* or detect* or assay* or diagnos*)) or radt or ridt or naat).mp.	5,796
**6**	or/1-5	9,026
**7**	exp influenza A virus/ or exp influenza B virus/ or exp influenza, human/	3,424
**8**	(influenza or flu).mp.	10,489
**9**	exp respiratory syncytial viruses/	243
**10**	(respiratory syncytial virus* or RSV).mp.	1,360
**11**	exp SARS-CoV-2/ or exp COVID-19/	4,254
**12**	(nCoV* or 2019nCoV or 19nCoV or COVID19* or COVID or SARS-COV-2 or SARSCOV-2 or SARS-COV2 or SARSCOV2 or SARS coronavirus 2 or Severe Acute Respiratory Syndrome Coronavirus 2 or Severe Acute Respiratory Syndrome Corona Virus 2 or ((new or novel or "19" or "2019" or Wuhan or Hubei or China or Chinese) adj3 (coronavirus* or corona virus* or betacoronavirus* or CoV or HCoV))).mp. [mp=title, original title, abstract, floating sub-heading word, mesh headings, heading words, keyword]	15,861
**13**	exp respiratory tract infections/	22,969
**14**	(respiratory adj3 (infect* or virus* or viral)).mp.	14,108
**15**	or/7-14	49,566
**16**	6 and 15	1,055
**17**	limit 16 to yr=2010 - current	899
**18**	limit 17 to english	887
