# Supplementary figures and images for: Clinical impact of rapid molecular diagnostic tests in patients presenting with viral respiratory symptoms: A systematic literature review
Source: PLoS One. 2024 Jun 13;19(6):e0303560. doi: 10.1371/journal.pone.0303560 (PMC11175541; doi:10.1371/journal.pone.0303560)

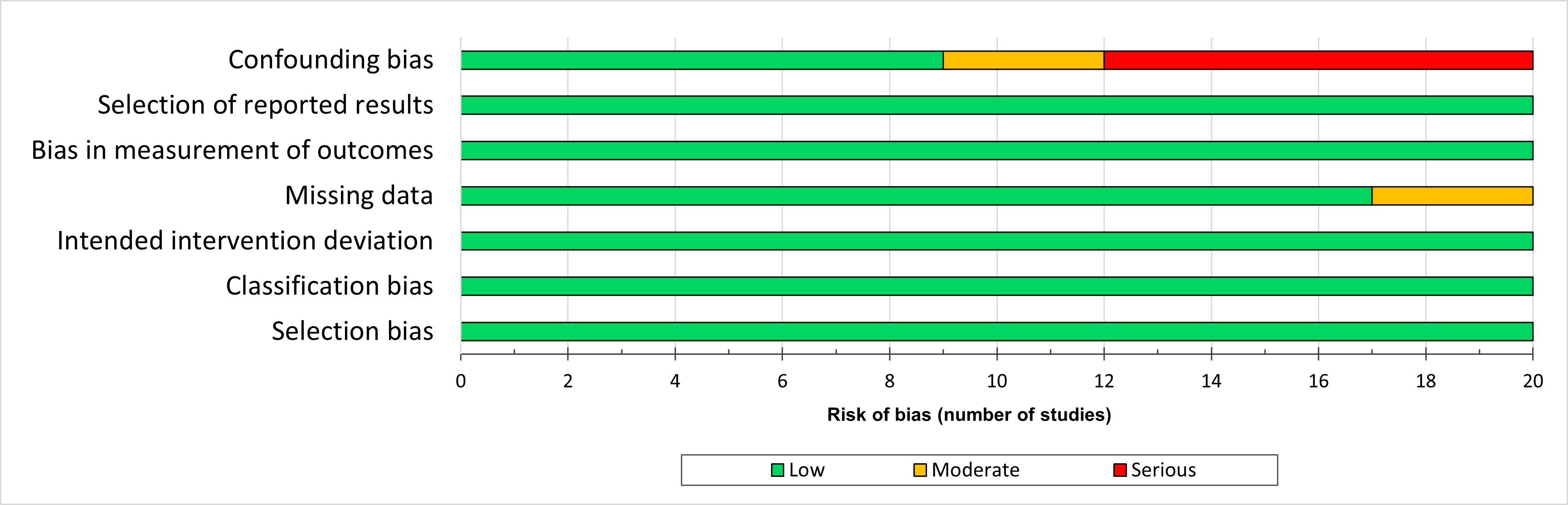

Supplement: S1 Fig — (TIF) [file pone.0303560.s005.tif]

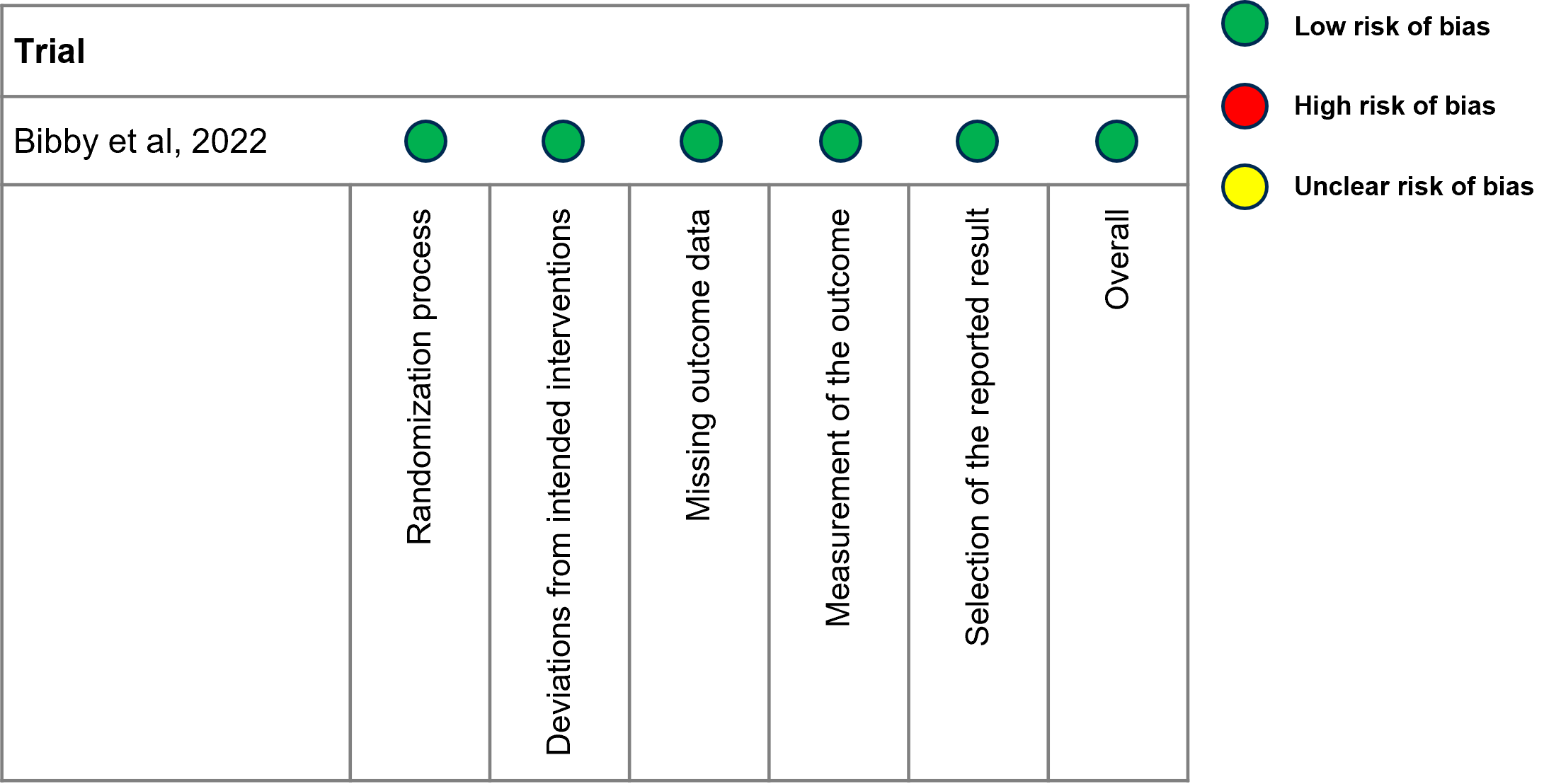

Supplement: S2 Fig — (TIF) [file pone.0303560.s006.tif]
